# Role of TNF α in the etiopathogenesis of PCOS: a clinical, biochemical and molecular genetic study

**DOI:** 10.1186/1755-8166-7-S1-P94

**Published:** 2014-01-21

**Authors:** Sujatha Thathapudi, Vijayalakshmi Kodati, Ahuja Yog Raj, Uma Addepally, Anuradha Katragadda, Qurratulain Hasan

**Affiliations:** 1 Vasavi Medical Research Centre, Hyderabad, India; 2 Jawaharlal Nehru Technological University, Hyderabad, India; 3 Anu Test tube baby centre, Hyderabad, India; 4 Kamineni Academy of Medical Sciences and Research Centre, Hyderabad, India; 5 Hyderabad Science Society, Hyderabad, India

## Background

Polycystic ovarian syndrome (PCOS) is one of the most common endocrine conditions affecting women of reproductive age with a prevalence of approximately 5-10 % worldwide. PCOS is characterized by hyperandrogenism, irregular menstrual cycles, polycystic ovaries, insulin resistance, obesity particularly abdominal phenotype. We evaluated the role of TNF α in PCOS patients and compared to age matched healthy controls.

## Methods

204 women clinically diagnosed with PCOS and 204 healthy women controls in the age group of 17 to 35 years were evaluated. PCOS were subdiveded into Obese and lean based on BMI (< 25 or ≤ >≥ 25 kg/m^2^). Clinical, biochemical characteristics of PCOS and molecular study of TNF alpha gene C850T polymorphism were studied.

## Results

Significant differences were observed in PCOS phenotypes and controls. All the PCOS including subtypes had elevated WC, W/H, Fasting Insulin, HOMA-IR, LH, LH/FSH, TG, and decreased HDL than Controls (P<0.0001). Patients had elevated serum TNF α, Free and Total testosterone, Androstenedione and DHEA when compared to controls (P <0.05). We have demonstrated an association between TNF α –C850T polymorphism and PCOS. Frequency of T allele was 93.5% in PCOS and 73.5% in controls (OR 5.1863, CI 3.2893 to 8.1773 and P value <0.0001). TT genotype confers a fivefold high risk for PCOS in our population (OR 5.7, CI 3.4673 to 9.3724 and P value <0.0001).

## Conclusion

TNF α contributes to the clinical , biochemical manifestations of PCOS, and –C850T TNF α gene polymorphism is associated with PCOS and could be used as a relevant molecular marker to identify women with risk of developing PCOS in our population.

